# Supplementary material for: The relevant resting-state brain activity of ecological microexpression recognition test (EMERT)
Source: PLoS One. 2020 Dec 22;15(12):e0241681. doi: 10.1371/journal.pone.0241681 (PMC7755225; doi:10.1371/journal.pone.0241681)
Supplement: Attachment — Submitted filename: renamed_67675.docx [file pone.0241681.s002.docx]

Please use the space provided to explain your answers to the questions above. You may also include additional comments for the author, including concerns about dual publication, research ethics, or publication ethics. (Please upload your review as an attachment if it exceeds 20,000 characters)

Reviewer #1: This manuscript used white, black and yellow models’ expressions as microexpressions and backgrounds to improve the materials ecological validity of EMERT, and used eyes-closed and eyes-open resting-state fMRI to detect relevant brain activity of EMERT for the first time. The main findings demonstrated there were many similarities and some differences of the relevant brain areas between microexpression M and SD.

I have several concerns that need to be addressed.

Answer: Thank you very much for your advice. We have revised the article according to your comments. Please see the blue font in the revised manuscript for details.

Main issues:

-About the EMERT’s retest reliability, which experimental results demonstrated that the EMERT had good retest reliability.

Answer: In the current study, the same participants were asked to do the same EMERT twice. Therefore the EMERT’s retest reliability could be measured by Pearson correlation between the two EMERT. It was found that each microexpression *M* in the first EMERT was significantly positively related to the corresponding one in the second EMERT and the *r*s (the plural of *r*, the same below) were high; and that each microexpression *SD* except surprise *SD* in the first EMERT was significantly positively related to the corresponding one in the second EMERT. These results showed that the EMERT had good retest reliability.

-The similarities and differences of the relevant brain areas of microexpression M and SD were not detailedly discussed in “Discussion” section, which tells readers less information.

Answer: Thank you very much! This advice was instructive. We have added the specific similarities and differences of the relevant brain areas of microexpression *M* and *SD*. Please see the blue font in 4.4 for details.

The microexpression *M* is the index of a microexpression type recognition. The microexpression *SD* is the background effect index of this microexpression type recognition (Yin, Tian, Hua, Zhang, & Liu, 2019; Zhang et al., 2017). The former is a kind of ability, but the latter is the degree that this ability changes in different contexts, which, in turn, can be thought of as the stability of this ability. Therefore, there should be similarities and differences in brain mechanisms between them.

In the eyes-closed resting state, ALFFs in the frontal lobe, insula, cingulate cortex, caudate nucleus, thalamus and vermis were significantly correlated with both some microexpression *M* and some microexpression *SD*, which indicates they need emotional perception and feeling. But ALFFs in hippocampal were only significantly correlated with both some microexpression *M*, which indicates that the microexpression type recognition ability need memory more; and ALFFs in the occipital lobe, parietal lobe, precuneus, putamen lobe, temporal lobe and cerebellum were only significantly correlated with some microexpression *SD*, which indicates that the stability of the microexpression type recognition ability need cognitive control, consciousness and motion more.

In the eyes-open resting state, ALFFs in the frontal lobe, insula, cingulate cortex, parietal lobe, caudate nucleus, thalamus, temporal lobe, cerebellum and vermis were significantly correlated with both some microexpression *M* and some microexpression *SD*, which indicates they need emotional perception and feeling. But ALFFs in hippocampal were only significantly correlated with both some microexpression *M*, which indicates that the microexpression type recognition ability need memory more; and ALFFs cuneus, occipital lobe, precuneus and putamen lobe were significantly correlated with some microexpression *SD*, which indicates that the stability of the microexpression type recognition ability need visual, consciousness and motion more.

In difference of eyes-open minus eyes-closed resting-states, ALFFs-difference in the frontal lobe, insula, amygdala, occipital lobe, fusiform, temporal lobe, cerebellum and vermis were significantly correlated with both some microexpression *M* and some microexpression *SD*, which indicates they need emotional perception and feeling. But ALFFs-difference in the cingulate cortex and parietal lobe was only significantly correlated with some microexpression *SD*, which indicates that the stability of the microexpression type recognition ability needs cognitive control more.

Taken together, both microexpression *M* and microexpression *SD* need emotional perception and feeling, but the former need memory more, and the latter need cognitive control and consciousness more. Of course, a certain ability requires its related brain areas and memory, but in addition to the brain areas associated with this ability, the stability of this ability requires cognitive control and consciousness. The similarities and differences in brain mechanisms of microexpression *M* and *SD* are logical. All these relevant brain areas can be trained to enhance ecological microexpression recognition ability.

-Do you have any criterion to choose the types of basic microexpressions or emotion model.

Answer: We chose the same six types of basic microexpressions as EMERT (Zhang et al., 2017), but the models must contain white, black and yellow people to improve the degree of ecologization for EMERT. So the criterion was as follows: Seven kinds of basic expression opened mouth pictures of eight models (four male and four female, including white, black and yellow people) from the NimStim face expression database (Tottenham et al., 2009) were used as the backgrounds, namely, neutral, sadness, fear, anger, disgust, surprise, and happiness. Except for neutral expression, the other six kinds of expressions were used as microexpressions. The pixels of all images were modified to be 338 × 434 with grey background (GRB: 127, 127, 127) (Zhang et al., 2017). Since each model in the NimStim face expression database had only two images for each expression, such as mouth-open and mouth-closed, we chose mouth-open with stable pleasure and arousal, so we were unable to measure different pleasure and arousal of a microexpression.

Minor issues:

-On page 5, need a reference for head motion correction.

-On page 7, is there any error for “A ingle sample t test”.

-On page 7, what does “rs” mean.

Answer:

-On page 5, a reference for head motion correction (adopting Friston 24) was added.

-On page 7, the correct spelling was “A single sample *t* test”.

-On page 7, “*r*s” means the plural of r, the same below.

Reviewer #2: I just three minor questions. One is that the ALFF in abstract should be provided the full name for the first time. The second one is that the referrences should be carefully checked. Third, the language should be edited by a native speaker.

Answer: Thank you very much for your advice. We have revised the article according to your comments. Please see the blue font in the revised manuscript for details. The ALFF in abstract was provided the full name “Amplitude of Low-Frequency Fluctuations” for the first time. The referrences were carefully checked and we added one referrence. The language was edited by a translation company.
